# 2023年毛细管电泳技术年度回顾

**DOI:** 10.3724/SP.J.1123.2024.02007

**Published:** 2024-05-08

**Authors:** Yuchen SHAO, Yalun WEN, Xinying ZHAO, Feng QU

**Affiliations:** 1.北京理工大学生命学院, 北京 100081; 1. School of Life Science, Beijing Institute of Technology, Beijing 100081, China; 2.北京电子科技职业学院, 北京 100176; 2. Beijing Polytechnic, Beijing 100176, China

**Keywords:** 毛细管电泳, 2023, 年度回顾, capillary electrophoresis (CE), 2023, annual review

## Abstract

为方便读者快速了解毛细管电泳(capillary electrophoresis, CE)技术在2023年的发展,根据国际通用学术水平评价指标之一的影响因子(IF)选择期刊,结合与CE技术紧密相关的实验类工作回顾了2023年的重要研究和应用进展。在ISI Web of Science数据库中通过主题检索所获得的2023年CE技术相关的669篇研究论文中,重点介绍了IF大于10.0的*Nature Communications*, *Nucleic Acids Research*, *Engineering*, *Journal of Medical Virology*和*Carbohydrate Polymers*发表的5篇实验类论文;IF为5.0~10.0的代表性期刊*Analytical Chemistry*, *Analytica Chimica Acta*, *Talanta*和*Food Chemistry*的31篇实验类论文;对IF小于5.0但CE技术报道较为集中的*Journal of Chromatography A*和*Electrophoresis*以及国内重要中文核心期刊(北京大学)中的代表性实验研究进行了概述。2023年,IF≥10.0期刊报道的最新科研进展都是使用已报道的CE方法,为CE技术的推广应用提供了新的突破口。此外,CE与质谱的新应用研究依旧是热点;CE在3D打印和水下工作系统等硬件方面的报道增加,在固体颗粒、细胞囊泡、细胞、病毒和细菌等非溶液样品分析中有较大突破,在药物和成分分析方面依旧保持优势。中文期刊方面,CE应用论文数量多于往年,尤其是在印刷领域的新应用十分抢眼。

## Abstract (en)

This paper serves as an annual review of capillary electrophoresis (CE) technology for 2023. The journals were selected based on their impact factor (IF), a universally recognized academic performance metric, combined with experimental work closely related to CE technology, to facilitate the rapid acquisition of significant research and application advancements in CE technology in 2023. A thematic search of the ISI Web of Science database yielded 669 research papers on CE technology published in 2023. This review highlights five experimental papers published in journals with IFs greater than 10.0, including *Nature Communications*, *Nucleic Acids Research*, *Engineering*, *Journal of Medical Virology*, and *Carbohydrate Polymers*, and 31 experimental papers from representative journals with IFs between 5.0 and 10.0, such as *Analytical Chemistry*, *Analytica Chimica Acta*, *Talanta*, and *Food Chemistry*. It also provides an overview of experimental research in journals with focused reporting on CE technology but with IFs less than 5.0, such as *Journal of Chromatography A* and *Electrophoresis*, as well as significant experimental research from key domestic Chinese core journals (Peking University). In 2023, all the latest scientific advancements reported in journals with an IF greater than 10.0 utilized previously reported CE methods, offering new breakthroughs for the promotion and application of CE technology. Additionally, new applications of CE in conjunction with mass spectrometry remained a hot topic. An increase in reports on the hardware aspects of CE, such as 3D printing and underwater systems, and significant breakthroughs in the analysis of non-solution samples, such as solid particles, cell vesicles, cells, viruses, and bacteria, was noted. CE is advantageous for the analysis of drugs and their components. In Chinese journals, the number of papers on CE applications exceeded that in previous years, with particular focus on the field of printing for new applications.

以“capillary electrophoresis-mass spectrometry”或“capillary isoelectric focusing”或“micellar electrokinetic chromatography”或“capillary electrophoresis”为关键词在ISI Web of Science数据库中进行主题检索(排除“capillary electrochromatography”“microchip”“microfluidic”“capillary monolithic column”),检索到时间范围为2023年1月1日至12月31日内的期刊论文共计669篇。11种影响因子(IF)≥10.0的期刊共计发表毛细管电泳(capillary electrophoresis, CE)相关的综述类论文28篇,实验类论文8篇;其中,*Nature Communications*(IF 16.6), *Nucleic Acids Research*(IF 14.9), *Engineering*(IF 12.8), *Journal of Medical Virology*(IF 12.7)和*Carbohydrate Polymers*(IF 11.2)分别发表了CE紧密相关的实验研究论文各1篇。10.0>IF≥5.0的84种期刊发表了CE相关论文145篇,*Analytical Chemistry*(IF 7.4)发表14篇,*Analytica Chimica Acta*(IF 6.2)发表12篇,*Talanta*(IF 6.1)发表8篇;影响力较大的食品分析期刊*Food Chemistry*(IF 8.8)发表2篇。IF<5.0的217种期刊发表了CE相关论文488篇,与CE关联密切的*Journal of Chromatography A*(IF 4.1)和*Electrophoresis*(IF 2.9)分别发表24篇和38篇。以“毛细管电泳”为关键词,在中国知网数据库检索到23种中文核心期刊(北京大学)发表了相关论文28篇。

本文根据国际通用学术水平评价指标之一——影响因子选择期刊,结合与CE技术紧密相关实验类工作进行介绍,便于读者快速了解CE技术在当年的重要研究和应用进展。

## 1 IF≥10.0期刊

2023年全年,IF≥10.0期刊发表了5篇和CE技术紧密相关的实验类论文,内容均属于已报道CE方法的新应用,分别来自2011年*Plant physiology*(IF 7.1)、2019年*SLAS Technology*(IF 2.7)、2020年*Journal of Chromatography A*(IF 4.1)和2021年*Analytical Chemistry*(IF 7.4)以及2015年出版的*Methods in Molecular Biology*。这些方法在当年的CE技术年度回顾中有相关介绍^[1,2]^。

### 1.1 CE-质谱(MS)

Marie等^[3]^在*Nature Communications*上发表了基于高灵敏度毛细管区带电泳(CZE)-MS深度分析生物样品中N-聚糖(N-glycans)的结果。在前期工作的基础上^[4]^,优化了CZE-MS的检测灵敏度和分离性能参数,开发了无标记N-聚糖分析的CZE-MS法。与传统CZE-MS方法相比,该方法使用样品量小于25 ng的模型蛋白和nL水平的血浆,信号强度增加了45倍,对纯化的人血清免疫球蛋白G、牛血清胎蛋白、牛胰腺核糖核酸酶B、血源细胞外囊泡(extracellular vesicle, EV)分离物和总血浆进行定性和定量分析,分别检测到250、400、150、310和520个以上的N-聚糖。与使用其他生物学方法测定的数量及其他复杂性相似的生物样本的检测结果相比,该方法测定的N-聚糖数量增加了大约15倍,同时还可以分析高度唾液酸化和未处理唾液酸的N-聚糖结构和异构体。

Cajic等^[5]^在*Engineering*上发表了可去除染料(removable dyes)结合多种方法深度分析N-聚糖缺失环节的研究结果,他们直接使用Hennig等^[6]^建立的八通道阵列CE,并创新性地使用9-氟氯甲酸甲酯染料作为可移动荧光标记,使多路毛细管凝胶电泳-激光诱导荧光检测法(xCGE-LIF)和基质辅助激光解吸/电离飞行时间质谱法(MALDI-TOF-MS)得以结合使用,从而实现更全面的N-糖基结构分析。并通过对鸡卵清蛋白、马血清和牛转铁蛋白中复合N-聚糖的详细结构分析,证明该方法“可视化”了迄今为止难以识别的人类免疫球蛋白A上的硫酸盐聚糖等N-聚糖,甚至包括N-聚糖结构的微小变化。

Katahira等^[7]^在*Nucleic Acids Research*上揭示了SARS-CoV-2中的Nsp14通过靶向定位核帽复合物抑制mRNA加工和核输出的过程,其中靶向CE-MS直接使用了Oikawa等^[8]^建立的用于组合单细胞代谢组学的CE-TOF-MS法,以1 mol/L甲酸背景电解质进行阳离子分析,以20 mmol/L甲酸铵(pH 10.0)为背景电解质进行阴离子分析,分别使用8 mmol/L甲基砜(用作阳离子分析)和樟脑磺酸(用于阴离子分析)的氯仿-甲醇(2∶5, v/v)溶液作为内标校正质谱峰面积。用上述方法检测Nsp14表达细胞产生的7-甲基三磷酸鸟苷,通过与已知结果对照估算表达4.3×10^6^个Nsp14的细胞产生的m7GTP为(954±117) pmol,转化成细胞浓度约为119 μmol/L。

### 1.2 CE

Jiang等^[9]^在*Journal of Medical Virology*上揭示了北京地区COVID-19非药物干预下儿童患者呼吸道合胞病毒感染流行模式的变化,直接使用了Li等^[10]^建立的基于CE的多重PCR法,利用八通道xCGE-LIF检测荧光标记的PCR产物,将其分离成14种长度的片段,通过和已知序列比对确认感染源类型,对所有样本的检测结果做系统进化分析,得到模式变化情况。Peluso等^[11]^在*Carbohydrate Polymers*发表了*γ*-环糊精(*γ*-cyclodextrin, *γ*-CD)和三甲基-*β*-CD形成达卡他韦复合物的分子建模研究结果,直接使用了Krait等^[12]^建立的CE方法,在未涂层石英毛细管中,检测波长为305 nm,以50 mmol/L的磷酸钠为背景电解质,并优化CE的分离温度和缓冲液pH,通过达卡他韦(daclatasvir, DCV)主要成分及其*R*,*R*,*R*,*R*构型对映体(*RRRR*-DCV)电泳峰之间的平台变化,考察复合物形成情况,结合等温滴定量热法测量数据构建数据模型。

## 2 IF<10.0的重要期刊

### 2.1 CE-MS分析技术

#### 2.1.1 蛋白质

Xu等^[13]^将高场不对称波形离子迁移谱与CE-MS结合,表征不同大小和电荷的蛋白质形式,尤其是针对质量范围20~45 kDa的较大蛋白质的形态数量鉴定,比单独使用CZE-MS/MS增加了6倍。Schlecht等^[14]^使用二维CZE-MS结合内酶切法,分离了完整的单克隆抗体(mAbs)电荷变体,在转移约640 pg的微小电荷变异体后,重链和轻链(HC+LC)组合序列覆盖率达到91.5%,具有很高的灵敏度。Wu等^[15]^将全柱成像毛细管等电聚焦(imaged capillary isoelectric focusing, icIEF)与MS耦合,分析mAbs蛋白质电荷变体的样本量仅为强阳离子交换法-MS的1/10即可达到相同的响应值,残留效应更低,鉴定准确度和分辨率更高。Vergara-Barberán等^[16]^利用在线适配体亲和固相萃取-CE-MS对牛奶中容易导致过敏的*β*-乳球蛋白进行纯化、预浓缩、分离、定性和定量,检出限(LOD)为0.05 mg/L,比CE-MS灵敏200倍,有望用于已有适配体的过敏原分析。Tomašovsk $\overset{'}{y}$ 等^[17]^建立了CZE和电喷雾离子源质谱(ESI-MS)联用检测注射液中胰岛素样生长因子-1(IGF-1)的方法,LOD为0.25 mg/L,可以用于IGF-1的检测。该方法展示了CE在药物质量控制中的潜力,与现行生物法相比,具有分离效率高、分析速度快、样品消耗量小的优势。

Wang等^[18]^利用CZE-MS/MS对小鼠脑中富集整合膜蛋白(IMPs)进行“自上而下”蛋白质组学分析,有效地鉴定和定量复杂蛋白质的混合物,证明了CZE-MS/MS在IMPs的蛋白质组学研究中有重要应用。Choi等^[19]^基于CE-ESI和捕获离子迁移谱-飞行时间质谱(trapped ion mobility spectrometry time-of-flight mass spectrometry, TimsTOF PRO)联用开发的微分析蛋白质组学技术,可以量化主要生物钟主导区域视交叉上核在光感受器依赖性视觉功能出现前后的发育变化。该技术是TimsTOF PRO在基于CE-ESI的微蛋白质组学中的首次系统应用,能够在单个细胞水平上观察并分析蛋白质表达的动态变化,揭示了生物钟发育中关键蛋白质的作用。

Sarkozy等^[20]^通过引入同轴鞘流反应器接口连接毛细管凝胶电泳仪和电喷雾离子化质谱仪,在没有显著离子抑制的情况下利用对质谱不友好的缓冲成分保持电喷雾过程的稳定,显著提高了CE-ESI-MS耦合的多功能性及分析肽和蛋白质的稳定性。

#### 2.1.2 脂质

Ly等^[21]^利用多段注射(multisegment injection,MSI)-非水CE(NACE)-MS定量测定了血液样本中的脂肪酸,该方法还可以在负离子模式下分辨其他类别的阴离子脂质,如磷脂酸和磷脂酰肌醇,并且能够利用ESI-MS中的时间信号模式识别进行严格的分子特征选择和脂质鉴定,从而实现非靶向筛查;同时通过引入新型磷脂甲基化策略,拓展了正离子模式下,通过MSI-NACE-MS进行分析的脂质组覆盖范围。

#### 2.1.3 代谢产物

Mever等^[22]^利用CE-MS表征斑马鱼幼鱼提取物中的有机离子代谢产物,在12只斑马鱼幼鱼的混合物中分析出超过70种内源性代谢物,能在1只斑马鱼幼鱼提取物中分析出29种内源性代谢物,可以用于研究应激时分泌的皮质醇的作用。Chen等^[23]^使用低流量CE-MS分析人类高密度脂蛋白(HDL)中的氧化1-棕榈酰-2-花生四烯酸-sn-甘油磷酰胆碱的10种长链和12种短链产物,在质量浓度2.5~100 mg/L范围内,相关系数(*R*^2^)为0.9918, LOD为1.52 mg/L,定量限(LOQ)为4.6 mg/L,用于健康人和尿毒症患者HDL分析的分辨率高于HPLC-MS技术。Ren等^[24]^分别用无鞘液CE-MS与LC-MS定量分析苦竹竹笋(*Pleioblastus amarus* (Keng))的糖、氨基酸等201种初级和次级代谢产物,结果表明无鞘液CE-ESI-MS在异构体鉴定、样品用量、极性和非极性代谢物检测等方面更有优势。

#### 2.1.4 其他

Błońska等^[25]^利用CE分离并收集尿液中的人葡萄球菌(*S. Staphylococcus hominis*)和大肠杆菌(*Escherichia coli*),分别使用MALDI-MS和TOF-MS鉴定,为识别生物样品中的细菌和病毒等病原体提供了基础。Zajda等^[26]^使用CE-电感耦合等离子体-MS/MS法,研究了模拟生理条件下Pt-DNA加合物的形成过程,有效地消除了光谱干扰,可以在生理条件下简单快速地检查DNA铂化。

### 2.2 CE硬件优化

#### 2.2.1 硬件技术

3D打印硬件 Wang等^[27]^制造了能同时进行电容耦合非接触式电导检测(capacitively coupled contactless conductivity detection, C^4^D)、紫外吸收(ultraviolet absorbance, UV)检测和LIF检测的三合一新型检测器,与CE联用检测荧光素时LOD达到1.3 μmol/L(C^4^D)、2.0 μmol/L(UV)和1 nmol/L(LIF),校准范围为0.01~500 μmol/L。Liu等^[28]^制造了集成C^4^D和毛细管冷却装置的高速CE设备,能有效降低焦耳热,改善样品堆积和峰分辨率,在25 s内完成阴离子和阳离子的检测,为危险或有毒物质的快速检测提供了潜在的解决方案。Itterheimová等^[29]^制造了全自动开源CE取样器,能同时处理14个不同样品,可以作为实验自制CE仪器的模块化组件使用。

水下工作系统 Drevinskas等^[30]^采用3D打印的聚碳酸酯管进行密封,开发了具备从样本采集到数据存储的完整功能且可以适应水下环境压力和温度条件的全自动CE-C^4^D系统,对水环境中的氨基酸、Ca^2+^和Mg^2+^的LOD均达到了5.2 μmol/L。Drevinskas等^[31]^开发了封闭式流通电极储液器自动CE系统,将毛细管进样端和电极做成封闭式储液池,液体可以持续流动,最大限度地减少试剂体积,减轻电解效果,该自动CE系统可以在25 kV下稳定运行60 min,且因CE常用背景电解质的性能与重力矢量方向无关,此系统解决了常用CE开放式储液器无法用于太空及水下作业的问题。

其他硬件 Atia等^[32]^开发了具有集成样品拭子提取功能的全自动便携毛细管电泳仪,无需手动预处理样品,能够在1 min内测定制药设备不锈钢表面的利多卡因,回收率为81.3%, LOD为0.13 μg/拭子。Ferreira Santos等^[33]^提出了基于转子-定子阀的CE流体动力进样方法,使进样和流体处理发生在毛细管的高电压侧,使CE仪器与毛细管接地侧的检测系统兼容,有望用于与电化学检测器和电喷雾电离质谱的耦合。

#### 2.2.2 毛细管内修饰

酶微反应器 Siebert等^[34]^基于酶在磁性纳米粒子MnFe_2_O_4_上的锚定作用,以壳聚糖和戊二醛为交联剂,通过钕磁铁的排列方式将酶固定在毛细管中作为新型的酶微反应器,测定乙酰胆碱酯酶(AChE)的米氏常数(*K*_m_)为1.12 mmol/L,可以高效、低成本筛选和评估AChE抑制剂。Li等^[35]^基于金属有机框架沸石咪唑酯骨架-8(zeolitic imidazolate framework-8)固定化酶微反应器开发了筛选天然产物中脂肪酶抑制剂的在线毛细管电泳法,测得脂肪酶的*K*_m_为2.75 mmol/L,低于离线测定的动力学常数,表明固定化酶与底物具有较高的亲和性,可以用于筛选具有潜在脂肪酶抑制活性的化合物。

涂层 Atia等^[36]^利用化学气相沉积法将毛细管内硅烷修饰为3-缩水甘油醚氧丙基三甲氧基硅烷(GPTMS),在pH 3~9范围内的电渗流降低明显。Dhellemmes等^[37]^利用连续多重离子聚合物层制备涂层毛细管并应用于碳酸酐酶等5种模型蛋白混合物的检测,极大地提高了分离效率和可重复性。Roca等^[38]^研究了聚烯丙基胺盐酸盐等物质作为毛细管涂层的最后一层时对肌红蛋白等4种模型蛋白质混合物分离的影响,测定了蛋白质的保留因子。Wang等^[39]^以磺胺嘧啶(sulfadiazine, SDZ)为模板分子,制备了分子印迹聚合物(molecularly imprinted polymer, MIP)包覆的毛细管,并引入聚(2-甲基-2-噁唑啉)(poly(2-methyl-2-oxazoline), PMOXA)减少非特异性吸附,利用表面分子印迹技术制备了SDZ-MIP-PMOXA涂层毛细管,在线富集测定了牛奶和鸡卵清中的微量SDZ,在质量浓度5.0~100.0 μg/L范围内线性良好,LOD为1.5 μg/L。

### 2.3 CE分析技术

#### 2.3.1 细胞外囊泡和细胞分析

Gao等^[40]^将二维离线耦合非对称流场流分离(asymmetrical flow field-flow fractionation, AF4)、大体积样品堆积(large-volume sample stacking, LVSS)和CE相结合,改善了AF4从高丰度基质中分离EV的效果,成功地从人血清中纯化和收集了EV,并进行了下游蛋白质分析。Ste $\overset{'}{c}$ 等^[41]^使用CE-LIF分析监测了柠檬汁中分离出的EV的均一性和异质性。与二喹啉甲酸法和纳米颗粒跟踪分析法对比结果表明,CE可以作为监测EV分离过程、评估分离的EV质量和纯度的全面工具。Obeid等^[42]^基于CE开发了用于考察EV大小特性的泰勒色散分析(taylor dispersion analysis)方法,能够在约7 nL样品体积下测量牛奶源EV的绝对尺寸,无需校准,重复性RSD<10%,可以对含量在2×10^14^EV/L以上的EV进行定量测定。Gordon等^[43]^建立了毛细管等速电泳(capillary isotachophoresis,CITP)分选并定量混合细胞样品中不同种类细胞数量的方法,利用溶解在样品中的离子间隔物在电泳时产生特定细胞迁移率的有效细胞峰,确定混合细胞样品中各组分的浓度;分析了含有变形链球菌(*S. mutans*)等3种细菌的混合样品,与血细胞计数法相比,准确度为1%~11%, RSD为1%~14%,为微生物污染测试和无菌测试等领域的细胞定量提供了新方法。

#### 2.3.2 病毒和细菌分析

Ramírez等^[44]^利用icIEF免疫法表征腺相关病毒蛋白质电荷异质性,对20 mg/L的HEK293T全细胞裂解物分析的灵敏度提高了90倍以上,LOQ为1.5×10^11^~2.1×10^11^VP/L,解决了细胞培养物上清液和细胞裂解物复杂样品中衣壳蛋白检测灵敏度不足的问题,无需纯化。Zhang等^[45]^建立了核酸链置换分离细菌的CE方法,将探针和适配体组成的DNA复合物与金黄色葡萄球菌等3种菌体混合,适配体可以与细菌特异性结合并释放探针,通过对探针的分离间接地用CE对细菌进行检测,在2.5 min内即可完成,LOD为4.20×10^9^~1.75×10^10^ 菌落形成单位/L。

#### 2.3.3 药物分析

核酸 Bchara等^[46]^利用CE-UV结合多元曲线分析-迭代最小二乘法快速分析i基序(intercalated motif, i-motif)折叠平衡随pH和温度的变化,为i-motif和其他复杂DNA结构的折叠提供了新见解。Xu等^[47]^初步建立了SARS-CoV-2和短串联重复序列(short tandem repeat)的CE共检测体系,在保证法医学DNA分析效率的前提下同时检测到SARS-CoV-2,用于提示SARS-CoV-2感染个体特征,缩小法医学案件调查范围,探索了CE检测RNA病毒的可能性。Hutanu等^[48]^用荧光标记的肽核酸作为亲和探针建立了CE无凝胶杂交分析法,用于寡核苷酸、mRNA疫苗或重组腺相关病毒等不同复杂程度核酸治疗药物的定性和定量分析,使用多探针时LOD可达到pmol级。

抗体 Tardif等^[49]^利用icIEF结合主成分分析和最小二乘-判别分析法,实现了pI 7~9、质量浓度0.5~1.5 g/L的4种治疗性mAbs的准确识别并将其归属到相应mAbs簇,可以排除“未知”mAbs并创建新簇。Auer等^[50]^使用葡聚糖右旋糖酐-硼酸盐溶液为筛分基质的十二烷基硫酸钠-毛细管凝胶电泳法,评估了稀释和超稀释基质中20~225 kDa的蛋白梯度(protein sizing ladder)和达雷木单抗(daratumumab)的完整和亚基形式(包括ngHC片段)的分离效果,并使用Ferguson图和爬行图阐明了分离机制。Puerta等^[51]^开发了分析免疫球蛋白A(IgA)单体(mIgA)和分泌型IgA(sIgA)的CGE方法,展示了使用商业CGE试剂盒对两类IgA分析的可行性,并开发了用于IgA分析的新型凝胶缓冲液。Kumar等^[52]^以mAb作为模型样品,在单次CE运行中利用CE的死时间分析了3份mAb样品,与传统的单次CE-UV相比,总分析时间减少了77%,生产率提高了300%,且不影响分辨率及相对峰面积,重现性良好,有望进一步减少CE分析时间。Andrasi等^[53]^建立了CZE分析化学参数相似、但脱酰胺化过程不同的3种胰岛素的方法,能够分离出仅相差0.984 Da、不同程度脱酰胺化形式的胰岛素,甚至相同程度去酰胺形式(质量相同但形状略有不同)的胰岛素异构体,证明了CZE在分离重组人胰岛素及其脱酰胺化异构体方面的适用性。

手性 Xu等^[54]^以疏水低共熔溶剂(HDES)甲基三辛基氯化铵-辛酸(N8881Cl∶OctA)作为背景缓冲液中的新型伪固定相(pseudo-stationary phase),与CD联用显著提高噻苯乙咪唑等4种模型药物的对映体分离度。Nan等^[55]^提出了可调重力介导的毛细管电泳法,以水溶性负离子磺酸丙基醚-*β*-环糊精聚合物作为手性选择剂,分离了普萘洛尔等5种手性药物,分离效率都有明显提升。Sung等^[56]^通过超声增强和表面活性剂辅助的分散液-液微萃取、CE场放大进样结合C^4^D检测,实现了5对吩噻嗪类药物及其对映体的快速分离和浓缩。该方法在1~150 nmol/L范围内,*R*^2^大于0.99, LOD为0.24~0.28 nmol/L。García-Cansino等^[57]^以CD和手性离子液体混合物作为手性选择剂,利用电动色谱法(electrokinetic chromatography, EKC)分离了依鲁替尼的2种对映体,并评估了对小型甲壳动物卵的毒性。Ioannou等^[58]^验证了双羧甲基-*β*-CD/DES系统,可以显著提高苯丙胺衍生物的手性分离效果。Alawadi等^[59]^发现了羧甲基化麦芽糖糊精比麦芽环糊精对曲马多等碱性药物的对映体具有更高的分辨率。Otin等^[60]^以二甲基-*β*-环糊精结合在线极性切换的大体积样品堆积预富集法,分析运动员禁用的莫瑞林等4种常见生长激素释放激素,LOD为75~200 mg/L。

#### 2.3.4 成分分析

血液样品 Dvo $\dot{r}$ ák等^[61]^提出了基于CE的自动化分析干血斑(DBS)的方法,利用CE压力系统实现目标物的萃取,样本通量为6个DBS/h,检测非甾体抗炎药和氨基酸的精密度RSD分别小于5.1%和12.3%。Ryšavá等^[62]^使用聚乙烯吡咯烷酮和羧甲基纤维素/氧化6-羧甲基纤维素复合材料制作了吸附剂用于采集DBS样品,可在CE进样瓶中直接处理DBS并进行检测。结合中空纤维液相微萃取-CE对模型酸性药物华法林等5种物质进行定量分析,重复性RSD优于8.1%,回收率为70%~99%。

尿液样品 Morav $\dot{c}$ ík等^[63]^建立了CE全自动高通量分析干尿斑(dried urine spot, DUS)中尿酸的方法,24 h内完成了240个DUS样本的全自动尿酸测定,在33.3~1200 μmol/L范围内线性关系的*R*^2^大于0.998,峰面积和迁移时间的RSD小于3.2%。Dvo $\dot{r}$ ák等^[64]^提出了CE-C^4^D和CE-UV测定DUS中肌酐、尿酸等内源性分析物和去甲替林(nortriptyline)等8种碱性和酸性药物外源性分析物的方法,与尿液样品相比洗脱效率为88%~100%,精密度RSD小于5.5%。de Oliveira Moreira等^[65]^以咪唑为内标物,使用在出口端进样并进行分离的CZE-UV法测定了100份尿样的肌酐含量,该方法的LOD为(28.5±17.8) mg/L,准确度为82.4%;与传统的Jaffe反应相比,提高了重复性和准确性,不破坏样品且可以重复使用,同时适用于血清,有望成为肌酐检测的替代方案。Opekar等^[66]^研制了用于电膜萃取的微型探针,与CE在线联用测定人尿中的肌酐和碱性氨基酸,灵敏度较未萃取前分别高4.9倍和2.6倍,可用于全天候测量,实用性强。

植物样品 Xia等^[67]^通过在毛细管内的动态衍生,建立了集成衍生分离黄酮类化合物的CE-LIF法,定量分析苜蓿植物中的黄酮类化合物以及颗粒化的苜蓿,LOD为0.92~35.46 nmol/L,回收率为80.55%~94.25%。Jin等^[68]^开发了新型的在线两步压力进样辅助堆积预富集方法,同时检测大米和干姜中的2,4,5-涕丙酸和2,4-二氯苯氧乙酸(2,4-D),线性关系的*R*^2^在0.9986~0.9996范围内,灵敏度增强因子为85~97。Zhang等^[69]^基于分析质量设计(analytical quality by design, AQbD)优化了CE-光电二极管阵列检测器对秦艽药材(*Gentianae Macrophyllae Radix*, RGM)中4种环烯醚萜类化合物(iridoid compounds)的分离条件,LOD为2.32~9.58 mg/L,为AQbD在天然产物分析中的应用提供了参考。Zhang等^[70]^采用近红外光谱结合偏最小二乘回归(partial least squares regression, PLSR)算法构建以落干酸等3种物质为RGM质量指标的质量校正模型,转换后的CE指纹图谱与真实的指纹图谱基本吻合,6个主峰均能准确预测,可用于RGM的质量控制。

动物样品 Kvasni $\dot{c}$ ka等^[71]^通过分析样品酸性水解后产生的葡糖胺,开发了在线耦合CITP-CZE-电导检测分析昆虫甲壳素的方法。将28个昆虫样品的壳聚糖含量结果与文献数据对比,证明该法实验结果与文献数据相当,LOD为0.06 μmol/L, LOQ为0.2 μmol/L。Tie等^[72]^利用CE-LIF表征了游离多柔比星(doxorubicin, DOX)和多柔比星脂质体(liposomal doxorubicin, L-DOX)在大鼠体内的药代动力过程。结果显示随着总DOX浓度逐渐降低,L-DOX浓度处于稳定水平,24 h内质量浓度为26.8~36.4 mg/L。

环境样品 Liu等^[73]^利用UV-发光二极管诱导光反应在毛细管内直接将硝酸盐转化为亚硝酸盐,利用CE-C^4^D测定了亚硝酸盐浓度,LOD为13 μmol/L,可用于湖泊水样中总氮的测定。Yang等^[74]^建立了磁性多孔碳固相萃取结合CE测定双酚A等4种双酚类物质的方法,LOD为0.71~1.65 mg/L,为环境样品中双酚类化合物的测定提供了新方法。

食物样品 Ashmore等^[75]^开发和验证了CE-UV快速测定葡萄酒和苹果酒中游离SO_2_的方法,与自动滴定法、曝气氧化法和离散分析器法的结果相比,CE-UV可以准确测量各种葡萄酒和苹果酒样品中的游离SO_2_, LOD分别为0.5 mg/L和0.8 mg/L。

#### 2.3.5 其他

Kitagishi等^[76]^基于太赫兹时域光谱分析方法(terahertz time-domain spectroscopy, THz-TDS),利用砷化镓(GaAs)半导体基片作为“局部”太赫兹发射器将THz-TDS与CE相结合,开发了在线检测低相对分子质量羧酸的局部太赫兹发射光谱方法(CE-THz-TDS),可以系统地分析氢键等分子间/分子内弱相互作用力。Kne $\dot{z}$ evi $\overset{'}{c}$ 等^[77]^利用亲和毛细管电泳(affinity capillary electrophoresis, ACE)研究四价钒和五价钒与丁二酸在不同pH和不同浓度下的相互作用,ACE分别用于四价钒和五价钒的同时平衡,与传统方法相比得到了类似的稳定常数和精度。该方法减少了测定时间,在处理危险材料或少量配体时有重要作用。Hajduk等^[78]^分别用内标法和经典法CE测定吡啶、咪唑和肟类化合物的亲电常数(p*K*_a_),内标法测定p*K*_a_为3.0~10.5的酸和4.0~12.0的碱更为快速,经典CE适用于其他p*K*_a_范围的酸和碱,可以作为额外收集或预测p*K*_a_的工具。Jing等^[79]^基于毛细管反向电极极性堆叠模式建立了二维离线耦合非对称场流分离结合CE(AF4×CE)分析纳米颗粒以及亚微米胶体分散体系的方法,用于分离50、100、200、300和400 nm的聚苯乙烯颗粒组成的亚微米颗粒混合物,解决了紫外吸收随颗粒直径增加导致的CE-UV检测灵敏度低的问题,克服了大颗粒紫外吸收低的困难,灵敏度增强10~60倍。

## 3 中文期刊

《色谱》 张含智等^[80]^基于CE-ESI-MS和NACE分离模式,实现了舒尼替尼等5种酪氨酸激酶抑制剂的基线分离,获得稳定的质谱信号,绝对检出限达到amol级,灵敏度高于水相分离条件下的CE-MS。门雪等^[81]^以2,3-萘-二甲醛为标记试剂,建立了测定肝癌细胞(HepG2)中谷胱甘肽含量的CE-LIF法,在0.01~20.00 mmol/L范围内线性关系良好,LOD为0.006 μmol/L, LOQ为0.020 μmol/L。江若可等^[82]^以十二烷基三甲基溴化铵(DTAB)为电渗流反转剂,建立了同时测定消毒洗手液等日化品中三氯生等3种化合物的NACE-UV检测法,在1~100 mg/L质量浓度范围内,*R*^2^均大于0.99, LOD为0.2 mg/L, LOQ为1.0 mg/L。

《印染》 刘晓磊等^[83]^开发了定量测定不同结构活性黑5(SES-SES-black-5)等活性染料及其水解产物浓度的CE-UV方法,通过计算水解速率验证实际印染过程中活性染料中活性基的活性,用于优化印染工艺。晏栖云等^[84]^开发了同时测定纺织物中6种荧光增白剂的CE-C^4^D检测方法,在10 min内实现了基线分离,LOD为0.33~0.46 μg/L,加标回收率为95.8%~101.8%, RSD小于3.8%。韦笑笑等^[85]^将液相微萃取和EKC结合,建立了纺织品中23种芳香胺的快速检测方法,目标物能够在14 min内实现分离,LOD为0.2~1.6 mg/kg, LOQ为0.8~5.8 mg/kg,能够满足纺织品中禁用偶氮染料的大批检测需求。

其他 孙雪峰等^[86]^建立了高效CE-二极管阵列检测(DAD)同时测定淡水鱼肉中阿莫西林和氨苄西林残留的方法,在20 min内实现基线分离,LOD分别为20 μg/kg和30 μg/kg, LOQ分别为30 μg/kg和40 μg/kg。张春莉等^[87]^建立了CZE-DAD检测金银花中8种有机酸的方法,17 min内实现了基线分离,加标回收率为92.6%~106.8%, RSD小于5.0%。江凤浩等^[88]^合成了温敏性*N*,*N*-二甲基丙烯酰胺/*N*-异丙基丙烯酰胺无规共聚物(P(DMA-co-NIPAM)),并与聚*N*,*N*-二甲基丙烯酰胺共混开发了无胶筛分CE,对大片段DNA的分辨率明显提高。

## 4 结论

纵观全年,英文期刊方面,IF≥10.0期刊报道的最新科研进展都是对已报道方法进行了新应用,为CE技术的推广应用提供了新的突破口。CE-MS新应用依旧是研究热点,CE在药物分析方面依旧保持优势。此外,CE在硬件方面的报道大量增加,如3D打印和水下工作系统等;在分析非溶液样品如固体颗粒、细胞囊泡、细胞、病毒和细菌等分析上取得了较大突破。中文期刊方面,CE-MS、CE-LIF和CE-UV的应用均有报道且数量多于往年,尤其在印刷领域的新应用备受瞩目。

以上内容难免有遗漏和不妥之处,请广大学界同仁及应用从业人员批评指正。
